# Myosteatosis and muscle loss impact liver transplant outcomes in male patients with hepatocellular carcinoma

**DOI:** 10.1002/jcsm.13554

**Published:** 2024-08-27

**Authors:** Di Lu, Zhihang Hu, Hao Chen, Abid Ali Khan, Qingguo Xu, Zuyuan Lin, Huigang Li, Jianyong Zhuo, Chiyu He, Li Zhuang, Zhe Yang, Siyi Dong, Jinzhen Cai, Shusen Zheng, Xiao Xu

**Affiliations:** ^1^ Department of Hepatobiliary & Pancreatic Surgery and Minimally Invasive Surgery Zhejiang Provincial People's Hospital, Affiliated People's Hospital of Hangzhou Medical College Hangzhou China; ^2^ NHC Key Laboratory of Combined Multi‐organ Transplantation Hangzhou China; ^3^ Zhejiang University School of Medicine Hangzhou China; ^4^ Organ Transplantation Center Affiliated Hospital of Qingdao University Qingdao China; ^5^ Key Laboratory of Integrated Oncology and Intelligent Medicine of Zhejiang Province Hangzhou China; ^6^ Department of Hepatobiliary and Pancreatic Surgery Shulan (Hangzhou) Hospital Hangzhou China; ^7^ Department of Hepatobiliary and Pancreatic Surgery, First Affiliated Hospital Zhejiang University School of Medicine Hangzhou China; ^8^ Institute of Translational Medicine Zhejiang University Hangzhou China

**Keywords:** Muscle mass, Muscle radiodensity, Deceased donor liver transplantation, Liver cancer

## Abstract

**Background:**

Sarcopenia is associated with unfavourable long‐term survival in patients undergoing liver transplantation (LT) for hepatocellular carcinoma (HCC). However, the impact of myosteatosis and muscle loss on patient prognosis has not been investigated.

**Methods:**

Seven hundred fifty‐six HCC patients who received LT at 3 transplant centres were included. Computed tomography (CT) images of recipients were collected to measure skeletal muscle index (SMI) and skeletal muscle radiodensity (SMRA). The impact of myosteatosis on the prognosis of sarcopenic and non‐sarcopenic patients was studied separately. Muscle status was evaluated based on the presence of sarcopenia and myosteatosis. The muscle loss of 342 males was calculated as the relative change of SMI between pre‐ and post‐LT evaluations. Cox regression models were used to identify predictors of overall survival (OS) and recurrence‐free survival (RFS).

**Results:**

The study comprised 673 males and 83 females. The median follow‐up time was 31 months (interquartile range, 19–43 months). Prior to LT, 267 (39.7%) and 187 (27.8%) males were defined as sarcopenic (low‐SMI) and myosteatotic (low‐SMRA), respectively. For sarcopenic recipients, the presence of myosteatosis was followed by a 23.6% decrease in 5 year OS (*P* < 0.001) and a 15.0% decrease in 5 year RFS (*P* = 0.014). Univariate and multivariate analyses revealed that muscle status was an independent predictor of OS [hazard ratio (HR), 1.569; 95% confidence interval (CI), 1.317–1.869; *P* < 0.001] and RFS (HR, 1.369; 95% CI, 1.182–1.586; *P* < 0.001). Postoperatively, a muscle loss >14.2% was an independent risk factor for poor OS (HR, 2.286; 95% CI, 1.358–3.849; *P* = 0.002) and RFS (HR, 2.219; 95% CI, 1.418–3.471; *P* < 0.001) in non‐sarcopenic recipients (*N* = 209).

**Conclusions:**

Pre‐transplant myosteatosis aggravated the adverse impact of sarcopenia on liver transplant outcomes in male HCC patients. Post‐transplant muscle loss might assist in prognostic stratification of recipients without pre‐existing sarcopenia, intriguing new insights into individualized management.

## Introduction

Hepatocellular carcinoma (HCC) is the third leading cause of tumour‐related death worldwide, accounting for 70–85% of primary liver cancers.^1^, ^2^, ^3^ Liver transplantation (LT) is a life‐saving intervention for selected HCC patients. Along with the proposal of a series of candidate selection criteria,^2^, ^4^ sustained efforts have been made to achieve a better prognosis evaluation of patients receiving LT due to HCC. Although most of the previous studies focused on tumour biological behaviour for prognostic prediction, increasing evidence suggested that it is necessary to take patients' nutritional and metabolic status into consideration.^5^, ^6^, ^7^

As an important component of the human body, skeletal muscle can reflect patients' metabolic and nutritional status, showing significant correlations with patient outcomes in multiple diseases.^8^, ^9^, ^10^, ^11^, ^12^ Computed tomography (CT) has emerged as a non‐invasive clinical tool for assessing the mass and radiodensity of skeletal muscle.^13^, ^14^ A recent global study suggests that sarcopenia is associated with adverse survival in patients with HCC after LT beyond the Milan criteria.^15^ However, little is known about the prognostic value of myosteatosis in LT for HCC. Also, most current studies focus on the pre‐transplant status of skeletal muscle without giving much attention to the peri‐transplant changes in muscle mass. Therefore, we aimed to investigate the impact of myosteatosis and muscle loss on outcomes among patients transplanted for HCC.

## Methods

### Study population

**Figure 1:**
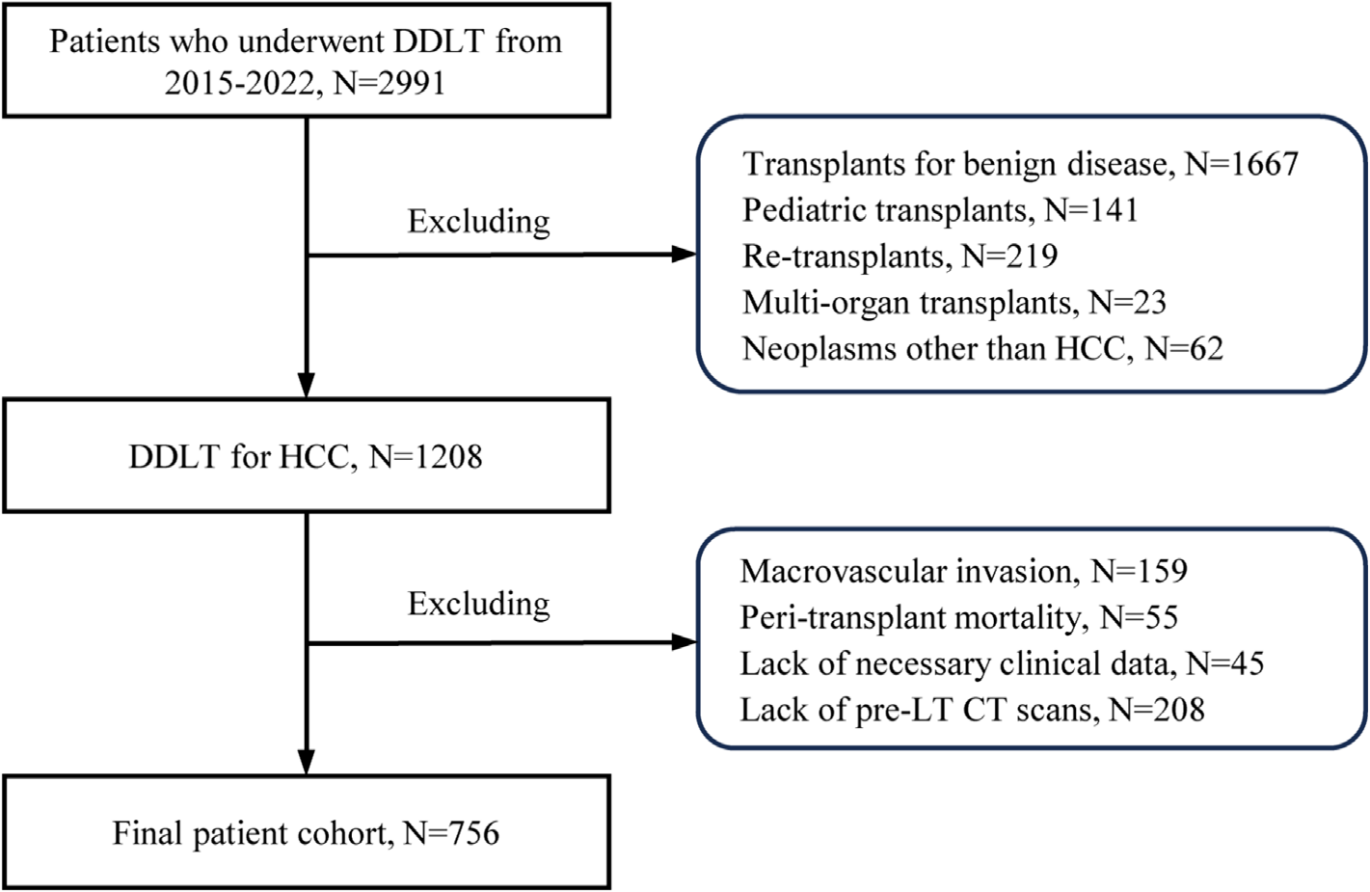
Flowchart of patient selection procedures. CT, computed tomography; DDLT, deceased donor liver transplantation; HCC, hepatocellular carcinoma; LT, liver transplantation.

This retrospective multicentre study included 2991 patients who received deceased donor LT (DDLT) for liver cancers in the First Affiliated Hospital of Zhejiang University School of Medicine (Hangzhou, China) between January 2015 and January 2021, Shulan (Hangzhou) Hospital (Hangzhou, China) between July 2017 and January 2021, and the Affiliated Hospital of Qingdao University between January 2015 and January 2022. The subject selection process is shown in *Figure* *1*. After excluding transplants for benign disease (*N* = 1667), paediatric transplants (*N* = 141), re‐transplants (*N* = 219), multi‐organ transplants (*N* = 23), transplants for neoplasms other than HCC (*N* = 62), patients with macrovascular invasion (*N* = 159), patients who died within 30 days after transplantation (*N* = 55), patients with incomplete clinical data (*N* = 45) and patients without pre‐LT CT scans (*N* = 208), 756 patients were studied. Out of the 756 patients, 342 males were selected for postoperative skeletal muscle assessment. The selection criteria were described subsequently. The clinical characteristics of the recipients chosen for dynamic analysis are shown in *Table* *S1*.

### Data collection

The clinical and anthropometric parameters were collected from the China Liver Transplant Registry (CLTR) strictly according to the Regulations on Human Organ Transplant and national legal requirements. Recipient characteristics included age, gender, body mass index (BMI), hepatitis B infection, the model for end‐stage liver disease (MELD) score before LT, Child–Pugh class, ascites, variceal bleeding, hepatic encephalopathy and pre‐transplant α‐fetoprotein (AFP). Tumour size and number were evaluated according to the pre‐transplant imaging, while the tumour differentiation grade and vascular invasion were histologically confirmed by post‐transplant pathology. The ABO compatibility of the donor and recipient was evaluated. Pre‐treatments of transarterial chemoembolization (TACE), liver resection and radiofrequency ablation (RFA) were included. Transplant characteristics included operative time, blood loss during operation and cold ischaemia time. The length of intensive care unit stay and the early postoperative complications, including bile leakage, biliary stricture, pulmonary infection and ascites, were documented and summarized by the CLTR. The survival statistics were updated according to a regular clinical follow‐up by the CLTR. The primary endpoint was recurrence or death due to any cause.

Abdominal CT images were retrospectively collected from the imaging departments of the three transplant centres. CT images used for measurement were all non‐enhanced with a voltage of 120 kV and slice thickness of 5 mm. Most transplant patients have undergone routine CT scans as part of their preoperative assessment, with some individuals excluded from the queue due to having only undergone MRI. The preoperative CT should be completed within 3 months before the transplant. In cases where a patient underwent multiple CT scans within this timeframe, the one closest to the surgery date was selected. After transplantation, recipients were followed by regular clinical evaluation and imaging examinations including a CT scan. However, due to the difference in follow‐up schemes among transplant centres, there is no exact time point for postoperative CT examination. In addition, a series of postoperative complications including unexplained abdominal pain, infectious or high fever, severe liver function abnormalities, structural abnormalities suggested by ultrasound, gastrointestinal bleeding and severe ascites require additional CT examinations. We included CT scans performed between postoperative day (POD) 20 and POD 50 as the subjects for analysis. Importantly, muscle mass and radiodensity changes over time during this period were not statistically significant according to our data (*Figure* *S1*). If a patient underwent multiple CT scans within this postoperative timeframe, the one closest to POD 30 was chosen.

### Skeletal muscle assessment

Skeletal muscle was evaluated at the third lumbar vertebra (L3) of cross‐sectional CT images using SliceOmatic software (version 5.0; Tomovision). The Hounsfield unit (HU) threshold of skeletal muscle was −29 to +150 HU as described previously.^16^ The muscle area was standardized for the patient's squared height in meters (m^2^) resulting in the skeletal muscle index (SMI). We used the cutoff values of SMI to define sarcopenia as described by Beumer *et al*.^15^: 45 cm^2^/m^2^ for men with a BMI < 25 kg/m^2^ and 51 cm^2^/m^2^ for men with a BMI ≥ 25 kg/m^2^. The mean skeletal muscle radiodensity (SMRA, in HU) was obtained from the aforementioned software to evaluate myosteatosis. In previous studies, low SMRA was defined as a mean attenuation <41 HU in patients with a BMI < 25 kg/m^2^ and <33 HU in those with a BMI ≥ 25 kg/m^2^.^17^, ^18^ However, considering the difference in BMI distribution between the global Eastern and Western populations (*Table* *S2*), we have refrained from using the previously proposed definition of myosteatosis. In addition, although the criteria of myosteatosis mentioned above were stratified by BMI categories, no statistical difference in SMRA was found in this study according to international and Chinese BMI categories (*Table* *S3*). Based on the current study population, myosteatosis was defined as SMRA less than 37.5 HU for men using maximally selected rank statistics. In dynamic analyses, muscle loss was calculated as the relative change in SMI after LT. Patients in this study's highest tertial of the muscle loss distribution (>14.2%) were considered to suffer high muscle loss. Similarly, using the same methodology, patients with a relative decrease in SMRA higher than 16.5% were considered to experience high SMRA reduction.

The measurement of skeletal muscle was conducted by two trained observers. Prior to the formal measurements, we randomly selected 50 CT images from the queue and provided them to the observers for assessing inter‐observer reproducibility. One week later, we had the two observers repeat the same tasks to evaluate intra‐observer reproducibility. The images of the aforementioned measurement results were validated by the anatomical radiologists. Before dynamic analyses, a precision test (30 CT images measured in duplicate by both observers) was performed to avoid random error caused by measurements according to a previously reported approach.^19^ Once the precision error of the measurements is known, the magnitude of the change in muscle that indicates real biologic change can be determined by the least significant change (LSC). The value with the larger error in the results of the two observers was used in the subsequent discussion.

### Ethics statement

The authors are accountable for all aspects of the work in ensuring that questions related to the accuracy or integrity of any part are appropriately investigated and resolved. The study design was strictly in accordance with the guidelines of the Regulations on Human Organ Transplantation and national legal requirements. Ethical approval for this study (reference number: NO. 20220021) was provided by the Institutional Review Board of the China Liver Transplantation Registration Scientific Committee. Informed consent was obtained. No organ was sourced from executed prisoners.

### Statistical analysis

Appropriate data type was chosen for each continuous and categorical variable listed in tables. Continuous variables were represented as median and interquartile range (IQR) or mean and standard deviation (*SD*), while numbers and percentages were used to describe categorical variables. Continuous univariates were compared with Student's *t*‐tests for normally distributed data and Mann–Whitney *U* tests for non‐normally distributed data. Chi‐squared or Fisher's exact test was used for categorical variables according to the sample scale. Precision error was reflected as the percentage coefficient of variation (%CV). The %CV was calculated as the root‐mean‐square coefficient of variation divided by the mean and expressed as a percentage. The 95% confidence LSC was defined for %CV precision error estimate by multiplying by 2.77 as mentioned in previous research.^20^ The study performed univariable survival analyses using Kaplan–Meier method and the stepwise Cox proportional hazards model to explore the prognostic significance for outcomes including overall survival (OS) and recurrence‐free survival (RFS). After univariate analyses, factors with *P* value <0.1 were taken into multivariate analyses. To distil the impact of preoperative muscle condition (skeletal muscle subgroup) on OS, 10 variables, namely, BMI, Child–Pugh class, ascites, MELD score, ABO compatibility, blood loss, preoperative AFP level, tumour size, tumour number and tumour differentiation were added in the multivariable regression; while to predict RFS, the 10 aforementioned variables and variceal bleeding were included in the multivariable regression. Furthermore, to determine the impact of muscle loss on OS, seven variables, namely, age, ABO compatibility, blood loss, preoperative AFP level, tumour size, tumour number, and tumour differentiation were included in the multivariable regression; similarly, the seven aforementioned variables and operative time were included in the multivariable regression to predict RFS. The variance inflation factors and tolerance values were calculated to evaluate the presence of statistically significant multicollinearity, with no significant collinearity detected. *P* values <0.05 were considered statistically significant. All the statistical analyses were performed using SPSS, version 28.0 (Chicago, IL, USA), and R software (version 4.2.2).

## Results

### Patient characteristics

The study comprised 756 patients (673 males and 83 females), and the median follow‐up time was 31 months (IQR, 19–43 months). The median recipient age was 53 (IQR, 47–59). The population exhibited a high prevalence of hepatitis B infection and cirrhosis, both at a rate of 91.7%. The median MELD score was 22 (IQR, 10–36). 381 (50.4%) patients were diagnosed with ascites while 92 (12.2%) experienced variceal bleeding. All recipients were confirmed postoperatively as HCC patients through pathological examination, and 372 (49.2%) of them met the Milan criteria. SMI and SMRA were measured based on pre‐LT CT images. The images were performed within 3 months before LT with a median interval of 19 (IQR, 4–29) days. The median SMI was 47.8 (IQR, 42.1–53.3) and the median SMRA was 40.7 (IQR, 36.2–44.5). Other baseline data, including recipient information and transplant characteristics, are shown in *Table* *1*.

**Table 1:** Patient characteristics at baseline in different populations

Characteristics	All (*N* = 756)	Sarcopenia (males)			Myosteatosis (males)		
		Yes (*N* = 267)	No (*N* = 406)	*P* value	Yes (*N* = 187)	No (*N* = 486)	*P* value
Males, *n* (%)	673 (89.0)	—	—	—	—	—	—
Age at transplant, median (IQR), year	53.0 (47.0–59.0)	54.0 (48.0–61.0)	53.0 (47.0–58.0)	**0.024**	56.0 (51.0–62.0)	52.0 (46.0–58.0)	**<0.001**
BMI, median (IQR), kg/m^2^	23.4 (21.5–25.8)	22.5 (20.8–25.3)	24.0 (22.5–26.5)	**<0.001**	23.9 (22.2–26.5)	23.4 (21.4–25.5)	**0.011**
Hepatitis B, *n* (%)	693 (91.7)	249 (93.3)	375 (92.4)	0.662	160 (85.6)	464 (95.5)	**<0.001**
Cirrhosis, *n* (%)	693 (91.7)	237 (88.8)	378 (93.1)	**0.050**	176 (94.1)	439 (90.3)	0.117
Ascites, *n* (%)	381 (50.4)	152 (56.9)	193 (47.5)	**0.017**	112 (59.9)	233 (47.9)	**0.005**
Variceal bleeding, *n* (%)	92 (12.2)	35 (13.1)	46 (11.3)	0.488	16 (8.6)	65 (13.4)	0.085
Hepatic encephalopathy, *n* (%)	24 (3.2)	11 (4.1)	9 (2.2)	0.155	5 (2.7)	15 (3.1)	0.778
MELD score, median (IQR)	22.0 (10.0–36.0)	24.0 (11.0–36.0)	23.0 (10.0–37.0)	0.365	24.7 (13.0–38.0)	23.1 (10.0–36.0)	0.124
Child–Pugh class, *n* (%)				**0.049**			**0.004**
A (5–6)	178 (23.5)	56 (21.0)	99 (24.4)		28 (15.0)	127 (26.1)	
B (7–9)	232 (30.7)	69 (25.8)	130 (32.0)		55 (29.4)	144 (29.7)	
C (10–15)	346 (45.8)	142 (53.2)	177 (43.6)		104 (55.6)	215 (44.2)	
Identical ABO compatibility, *n* (%)	694 (91.8)	243 (91.0)	373 (91.9)	0.695	169 (90.4)	447 (92.0)	0.504
Pre‐treatment, *n* (%)							
TACE	317 (41.9)	117 (43.8)	169 (41.6)	0.573	77 (41.2)	209 (43.0)	0.728
Resection	136 (18.0)	51 (19.1)	74 (18.2)	0.840	41 (21.9)	84 (17.3)	0.184
RFA	128 (16.9)	43 (16.1)	67 (16.5)	0.916	32 (17.1)	78 (16.0)	0.728
CIT, median (IQR), h	8.0 (5.6–9.3)	8.0 (5.3–9.1)	8.0 (5.9–9.5)	0.224	8.0 (6.5–9.9)	7.7 (5.4–9.2)	**0.002**
Operative time, median (IQR), h	5.7 (4.8–7.0)	5.8 (4.8–7.0)	5.7 (4.8–7.0)	0.416	5.8 (5.0–7.0)	5.7 (4.8–7.0)	0.439
Intraoperative blood loss > 1 L, *n* (%)	298 (39.4)	128 (47.9)	137 (33.7)	**<0.001**	100 (53.5)	165 (34.0)	**<0.001**
Tumour number > 3, *n* (%)	163 (21.6)	72 (27.0)	79 (19.5)	**0.022**	43 (23.0)	108 (22.2)	0.830
Sum of tumour diameters > 8 cm, *n* (%)	276 (36.5)	120 (44.9)	136 (33.5)	**0.003**	79 (42.2)	177 (36.4)	0.163
Last pre‐transplant α‐fetoprotein, *n* (%)				**0.006**			0.080
≤100 ng/mL	469 (62.0)	146 (54.7)	270 (66.5)		115 (61.5)	301 (61.9)	
100–400 ng/mL	94 (12.4)	38 (14.2)	50 (12.3)		17 (9.1)	71 (14.6)	
>400 ng/mL	193 (25.6)	83 (31.1)	86 (21.2)		55 (29.4)	114 (23.5)	
Tumour differentiation, *n* (%)				0.583			0.921
Complete necrosis/no viable tumour	70 (9.3)	19 (7.1)	41 (10.1)		18 (9.6)	42 (8.6)	
Well differentiated	42 (5.5)	14 (5.2)	23 (5.7)		9 (4.8)	28 (5.8)	
Moderately differentiated	406 (53.7)	149 (55.9)	214 (52.7)		99 (52.9)	264 (54.3)	
Poorly differentiated	238 (31.5)	85 (31.8)	128 (31.5)		61 (32.6)	152 (31.3)	
Meeting the Milan criteria, *n* (%)	372 (49.2)	108 (40.4)	211 (52.0)	**0.004**	84 (44.9)	235 (48.4)	0.439
Meeting the Hangzhou criteria, *n* (%)	591 (78.2)	188 (70.4)	335 (82.5)	**<0.001**	139 (74.3)	384 (79.0)	0.215
Meeting the UCSF criteria, *n* (%)	432 (57.1)	127 (47.6)	246 (60.6)	**<0.001**	99 (52.9)	274 (56.4)	0.437
SMI, median (IQR), cm^2^/m^2^	47.8 (42.1–53.3)	42.3 (38.4–44.8)	52.5 (48.7–57.3)	**<0.001**	46.3 (40.9–51.7)	49.6 (44.8–55.0)	**<0.001**
SMRA, median (IQR), HU	40.7 (36.2–44.5)	39.3 (35.2–48.0)	42.7 (38.7–45.4)	**<0.001**	34.6 (32.0–36.2)	43.5 (40.6–45.8)	**<0.001**
Sarcopenia, *n* (%)	—	—	—	—	108 (57.8)	159 (32.7)	**<0.001**
Myosteatosis, *n* (%)	—	108 (40.4)	79 (19.5)	**<0.001**	—	—	**—**
ICU stay, median (IQR), hours	201 (108–320)	207 (117–327)	208 (107–321)	0.338	206 (119–327)	208 (108–320)	0.150
Postoperative early complications, *n* (%)	124 (16.4)	46 (17.2)	61 (15.0)	0.444	37 (19.8)	70 (14.4)	0.087
1 year OS	87.8%	80.3%	92.3%	—	78.0%	91.3%	**—**
2 year OS	76.0%	64.3%	83.8%	—	65.1%	80.4%	**—**
3 year OS	69.2%	59.7%	75.2%	—	57.1%	73.6%	**—**

*Note*: Values in bold represent statistically significant differences.

Abbreviations: BMI, body mass index; CIT, cold ischaemia time; ICU, intensive care unit; IQR, interquartile range; MELD, Model for End‐Stage Liver Disease; OS, overall survival; RFA, radiofrequency ablation; SMI, skeletal muscle index; SMRA, skeletal muscle radiodensity; TACE, transarterial chemoembolization; UCSF, University of California, San Francisco.

### The clinical characteristics in male recipients with sarcopenia and myosteatosis

**Figure 2:**
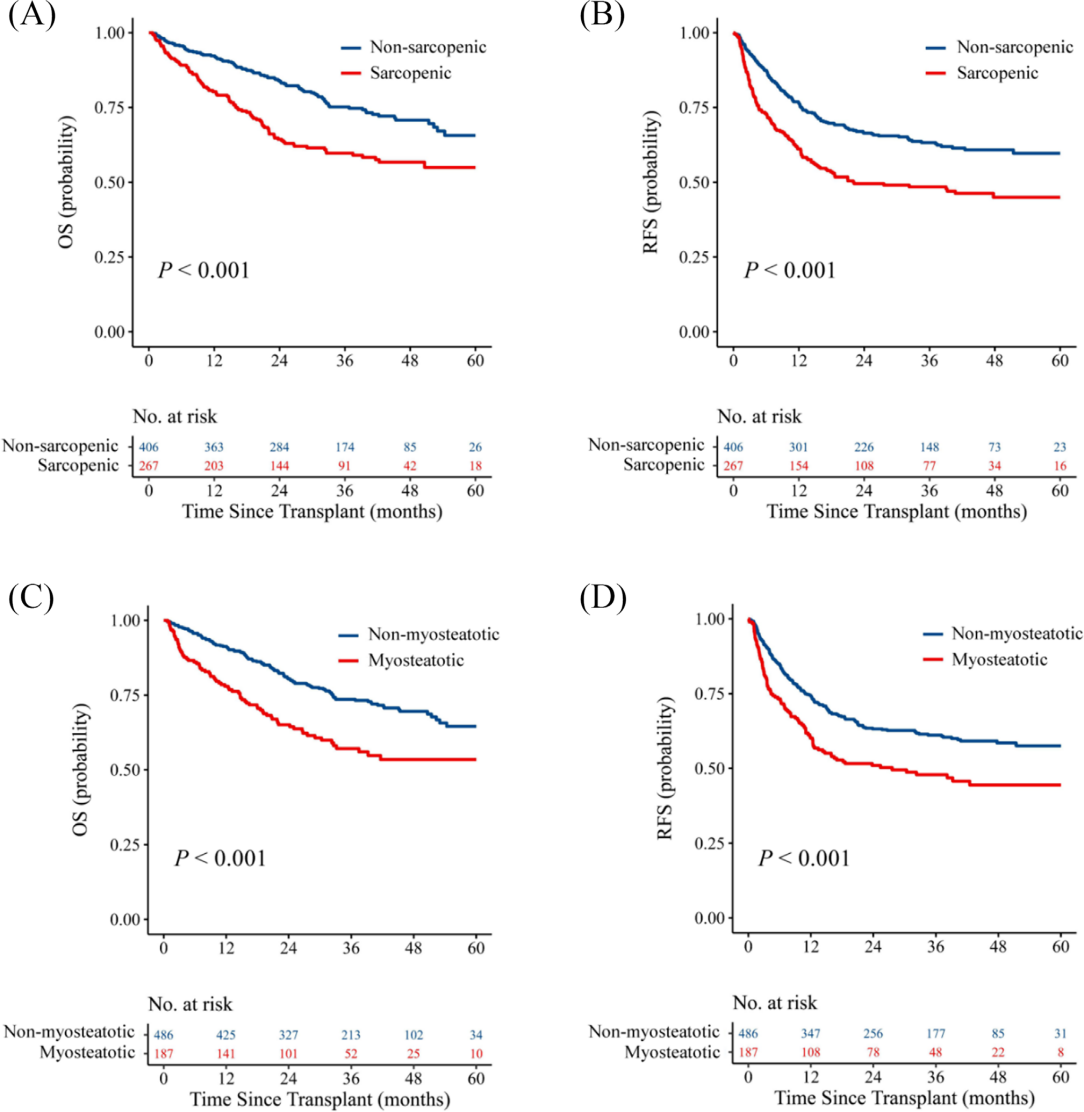
Survival curves for recipients with poor muscle status. (*A,B*) OS and RFS curves in recipients with or without sarcopenia using log‐rank tests. (*C,D*) OS and RFS curves in recipients with or without myosteatosis using log‐rank tests. CT, computed tomography; OS, overall survival; RFS, recurrence‐free survival.

Given the prognostic distinctions in body composition observed between genders (refer to *Table* *S4*), the following analyses were conducted exclusively within the male cohort. Among 673 males, 267 (39.7%) and 187 (27.8%) were classified as sarcopenic and myosteatotic, respectively. Recipients with sarcopenia and myosteatosis were older than those without (*P* = 0.024; *P* < 0.001). Sarcopenic recipients had a lower BMI than non‐sarcopenic recipients (*P* < 0.001) while myosteatotic recipients had a higher BMI than non‐myosteatotic recipients (*P* = 0.011). Among transplant factors, both sarcopenic and myosteatotic recipients showed an increased risk of intraoperative blood loss (*P* < 0.001). Regarding tumour characteristics, sarcopenic recipients had higher preoperative AFP (*P* = 0.006), tumour size (*P* = 0.003), and tumour number (*P* = 0.022) than non‐sarcopenic recipients. In terms of prognostic significance, sarcopenic recipients exhibited reduced OS (*P* < 0.001, *Figure* *2* *A*) and RFS (*P* < 0.001, *Figure* *2* *B*) compared with their non‐sarcopenic counterparts. Similarly, recipients identified with myosteatosis demonstrated decreased OS (*P* < 0.001, *Figure* *2* *C*) and RFS (*P* < 0.001, *Figure* *2* *D*) in comparison to those without myosteatosis. Other clinical characteristics of sarcopenic and myosteatotic recipients are presented in *Table* *1*.

### Sarcopenia and myosteatosis demonstrated overlapping prognostic values in male recipients

**Figure 3:**
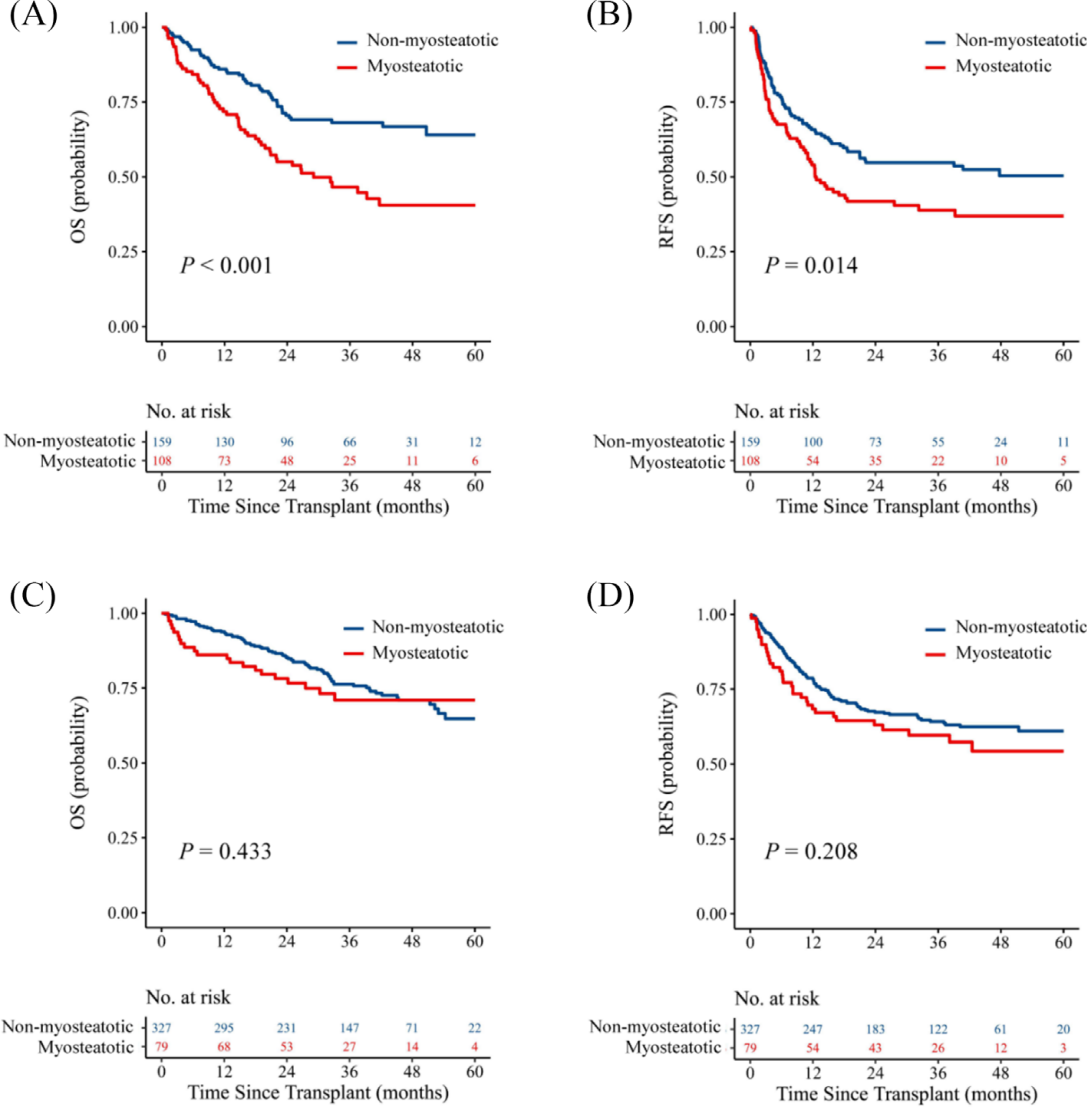
Survival curves for recipients with myosteatosis in the sarcopenic and non‐sarcopenic populations. (*A,B*) OS and RFS curves in the sarcopenic group with or without myosteatosis using log‐rank tests. (*C,D*) OS and RFS curves in the non‐sarcopenic group with or without myosteatosis using log‐rank tests. OS, overall survival; RFS, recurrence‐free survival.

Considering the notable comorbidity of sarcopenia and myosteatosis, the study conducted subgroup analyses to further investigate their contributions to prognosis. Within the sarcopenic subgroup, recipients with myosteatosis (*N* = 108) exhibited markedly reduced OS (*P* < 0.001, *Figure* *3* *A*) and RFS (*P* = 0.014, *Figure* *3* *B*) than those without myosteatosis (*N* = 159). Conversely, within the non‐sarcopenic subgroup, there was no significant discrepancy observed in either OS (*P* = 0.433, *Figure* *3* *C*) or RFS (*P* = 0.208, *Figure* *3* *D*) between recipients with myosteatosis (*N* = 79) and those without myosteatosis (*N* = 327). Based on the results of the subgroup analyses described above, the entire population was stratified into the following three skeletal muscle subgroups with different pre‐transplant muscle status: (a) poor (sarcopenia with myosteatosis); (b) intermediate (sarcopenia without myosteatosis); (c) good (non‐sarcopenia regardless of myosteatosis). The clinical characteristics of the above three groups of patients are presented in *Table* *S5*. Univariate and multivariate analyses confirmed that the pre‐transplant muscle status was an independent risk factors for adverse outcomes (OS: HR 1.569, 95% CI, 1.317–1.869, *P* < 0.001; RFS: HR 1.369, 95% CI, 1.182–1.586, *P* < 0.001). The MELD score, ABO compatibility, preoperative AFP level, tumour size, and tumour differentiation emerged as independent prognostic factors for both OS and RFS, as detailed in *Table* *2*. In addition, the high intraoperative blood loss is an independent risk factor for mortality rather than tumour recurrence. In summary, myosteatosis showed an additive effect on both primary endpoints among sarcopenic recipients, resulting in a compounded prognostic risk.

**Table 2:** Cox analyses associated with poor outcomes in male recipients (*N* = 673)

Variable	Univariable		Multivariable	
	HR (95% CI)	*P* value	HR (95% CI)	*P* value
Overall survival				
Age at transplant	0.996 (0.981–1.011)	0.598		
BMI	0.939 (0.898–0.983)	**0.006**	—	—
Cirrhosis	0.858 (0.535–1.375)	0.524		
Hepatitis B	0.957 (0.556–1.647)	0.874		
Child–Pugh class	1.295 (1.080–1.551)	**0.005**	—	—
Ascites	1.379 (1.046–1.820)	**0.022**	—	—
Variceal bleeding	0.761 (0.475–1.221)	0.257		
Hepatic encephalopathy	0.871 (0.359–2.117)	0.761		
MELD score	1.014 (1.003–1.025)	**0.013**	1.012 (1.000–1.023)	**0.049**
ABO compatibility	0.505 (0.357–0.714)	**<0.001**	0.500 (0.342–0.730)	**<0.001**
CIT	1.007 (0.961–1.055)	0.783		
Operative time	1.004 (0.929–1.086)	0.913		
Blood loss	1.768 (1.344–2.325)	**<0.001**	1.431 (1.076–1.905)	**0.014**
Preoperative AFP level	1.858 (1.601–2.156)	**<0.001**	1.564 (1.341–1.825)	**<0.001**
Tumour size	3.070 (2.321–4.060)	**<0.001**	2.103 (1.574–2.809)	**<0.001**
Tumour number	2.171 (1.625–2.900)	**<0.001**	—	—
Tumour differentiation	1.657 (1.361–2.018)	**<0.001**	1.437 (1.160–1.780)	**<0.001**
Muscle status^a^	1.669 (1.411–1.973)	**<0.001**	1.569 (1.317–1.869)	**<0.001**
Recurrence‐free survival				
Age at transplant	0.992 (0.979–1.005)	0.221		
BMI	0.943 (0.908–0.979)	**0.002**	—	—
Cirrhosis	0.731 (0.501–1.068)	0.105		
Hepatitis B	1.099 (0.690–1.750)	0.691		
Child–Pugh class	1.180 (1.016–1.371)	**0.031**	—	—
Ascites	1.232 (0.976–1.555)	**0.079**	—	—
Variceal bleeding	0.670 (0.448–1.003)	**0.052**	—	—
Hepatic encephalopathy	0.799 (0.377–1.692)	0.558		
MELD score	1.011 (1.001–1.020)	**0.026**	1.010 (1.000–1.019)	**0.046**
ABO compatibility	0.579 (0.425–0.787)	**<0.001**	0.619 (0.439–0.874)	**0.006**
CIT	1.008 (0.969–1.048)	0.697		
Operative time	1.001 (0.937–1.070)	0.969		
Blood loss	1.442 (1.143–1.819)	**0.002**	—	—
Preoperative AFP level	1.836 (1.618–2.083)	**<0.001**	1.497 (1.312–1.708)	**<0.001**
Tumour size	3.464 (2.732–4.392)	**<0.001**	2.574 (2.014–3.291)	**<0.001**
Tumour number	2.525 (1.979–3.222)	**<0.001**	—	—
Tumour differentiation	1.623 (1.381–1.908)	**<0.001**	1.380 (1.160–1.642)	**<0.001**
Muscle status^a^	1.478 (1.280–1.707)	**<0.001**	1.369 (1.182–1.586)	**<0.001**

*Note*: Values in bold represent statistically significant differences.

^a^ Muscle status was evaluated based on the presence of sarcopenia and myosteatosis as follows: (a) poor (sarcopenia with myosteatosis); (b) intermediate (sarcopenia without myosteatosis); (c) good (non‐sarcopenia regardless of myosteatosis).

Abbreviations: AFP, α‐fetoprotein; BMI, body mass index; CI, confidence interval; CIT, cold ischaemia time; HR, hazard ratio; MELD, Model for End‐Stage Liver Disease.

### Post‐transplant muscle loss impacted outcomes of non‐sarcopenic recipients

**Figure 4:**
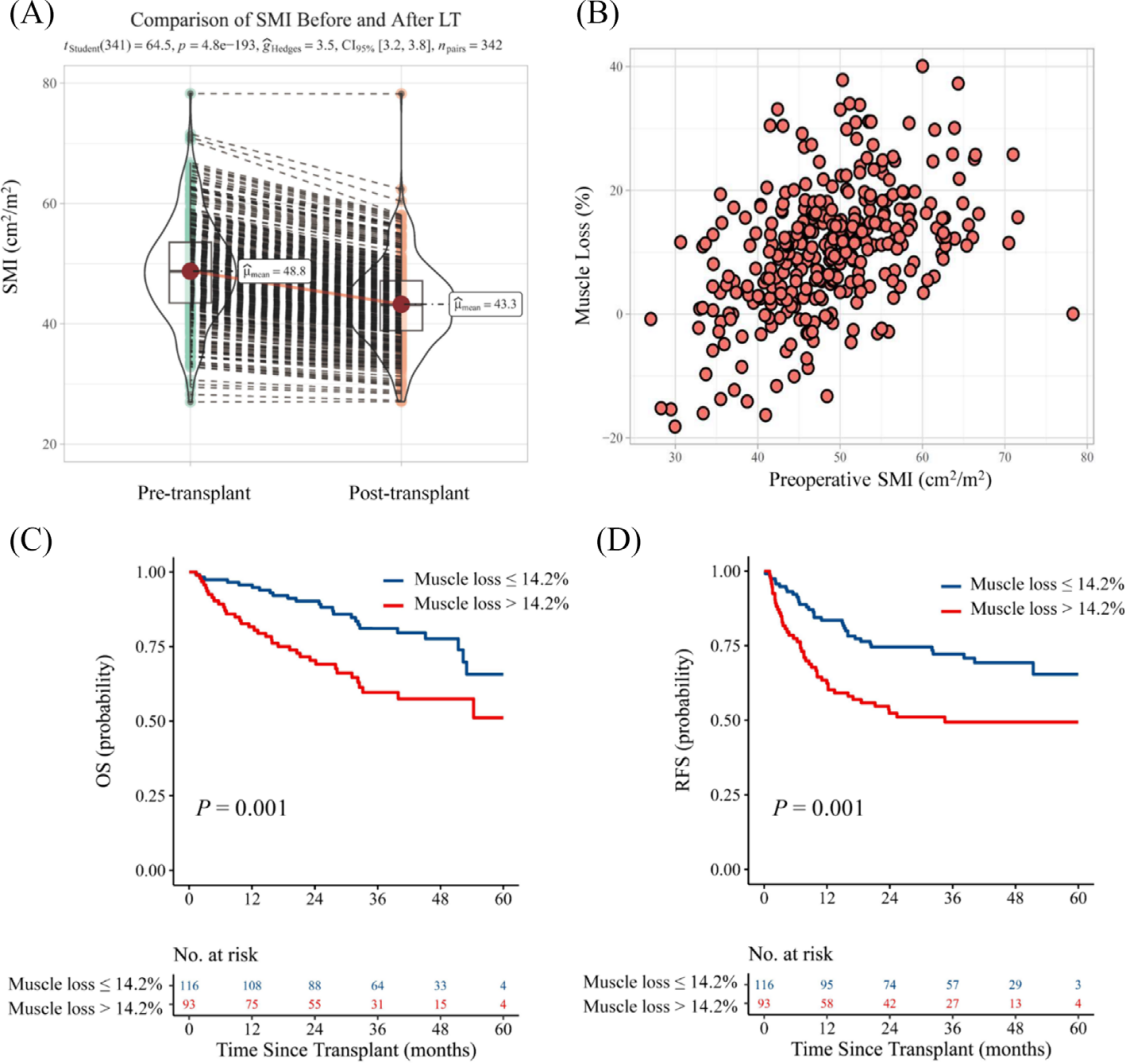
The variation of SMI and survival analyses of muscle loss. (*A*) The variation of SMI between the pre‐ and post‐LT evaluation using paired samples *t*‐test (*P* < 0.001). (*B*) The correlation between preoperative SMI and muscle loss using Pearson correlation analysis (*r* = 0.45, *P* < 0.001). (*C,D*) OS and RFS curves of non‐sarcopenic patients with muscle loss >14.2% or ≤14.2% using log‐rank tests. LT, liver transplantation; OS, overall survival; RFS, recurrence‐free survival; SMI, skeletal muscle index.

To investigate the impact of dynamic changes in muscle on patient outcomes, 342 males with available CT scans were analysed. All the CT scans were performed between POD 20 and POD 50, with a median interval of 30 (IQR, 24–35) days after LT. Correlation analyses revealed that the changes in SMI and SMRA did not progress over time from POD 20 to POD 50 (*Figure* *S1*), suggesting a period of relative stability. Compared with the total population, those patients with post‐transplant CT images showed no significant difference in tumour features, SMI, SMRA, early complication and 90‐day mortality. The precision error and the LSC of muscle measurements are presented in *Table* *S6*. The precision errors defined as %CV for skeletal muscle area and SMRA were 0.65% and 0.36%, respectively. The corresponding LSC_%CV_ values were 1.79% and 0.98%, respectively. Most recipients experienced a significant postoperative muscle loss as shown in *Figure* *4* *A*, with a median muscle loss of 11.3% (IQR, 4.5–16.2%). The correlation between preoperative SMI and muscle loss is depicted in *Figure* *4* *B*. SMRA also declined significantly after LT (*Figure* *S2*), with a median decrease of 12.4% (IQR, 4.1–18.8%).

We conducted separate analyses in both sarcopenic and non‐sarcopenic populations and noticed that 44.5% of non‐sarcopenic recipients suffered muscle loss >14.2%, while the ratio declined to 15.0% in sarcopenic recipients. Additionally, 34.4% of non‐sarcopenic recipients and 31.6% of sarcopenic recipients experienced a high SMRA reduction, respectively. Univariate and multivariate Cox analyses showed that muscle loss was an independent risk indicator for poor OS (HR, 2.286; 95% CI, 1.358–3.849; *P* = 0.002) and RFS (HR, 2.219; 95% CI, 1.418–3.471; *P* < 0.001) in non‐sarcopenic recipients (*Table* *3*) while no significant difference was observed in sarcopenic recipients (*Table* *S7*). In contrast, postoperative SMRA reduction showed no prognostic value in either of the two aforementioned subgroups. In the non‐sarcopenic population, recipients with muscle loss >14.2% (*N* = 93) had significantly worse OS (*P* = 0.001, *Figure* *4* *C*) and RFS (*P* = 0.001, *Figure* *4* *D*) than those without (*N* = 116). The clinical characteristics of the two populations are given in *Table* *S8*. Compared with the low muscle loss group, the high muscle loss group had higher age, intraoperative blood loss and lower ABO compatibility rate, although none of these factors showed a statistically significant difference. In summary, we found a significant decline in muscle mass at around 1 month after transplantation. Although non‐sarcopenic recipients appeared to have a favourable prognosis, the survival of a portion of patients who experienced muscle loss >14.2% did not meet our expectations.

**Table 3:** Cox analyses associated with poor outcomes in non‐sarcopenic patients (*N* = 209)

Variable	Univariable		Multivariable	
	HR (95% CI)	*P* value	HR (95% CI)	*P* value
Overall survival				
Age at transplant	0.970 (0.943–0.998)	**0.035**	—	—
BMI	0.960 (0.882–1.045)	0.350		
Cirrhosis	2.031 (0.496–8.321)	0.325		
Hepatitis B	0.824 (0.257–2.641)	0.745		
Child–Pugh class	1.160 (0.818–1.647)	0.405		
Ascites	1.307 (0.783–2.180)	0.305		
Variceal bleeding	0.802 (0.321–2.004)	0.637		
Hepatic encephalopathy	0.707 (0.098–5.113)	0.731		
MELD score	1.007 (0.986–1.028)	0.528		
ABO compatibility	0.394 (0.204–0.759)	**0.005**	—	—
CIT	1.024 (0.938–1.118)	0.600		
Operative time	1.100 (0.951–1.273)	0.199		
Blood loss	2.053 (1.233–3.417)	**0.006**	—	—
Preoperative AFP level	1.762 (1.329–2.337)	**<0.001**	—	—
Tumour size	3.585 (2.133–6.027)	**<0.001**	3.391 (2.009–5.722)	**<0.001**
Tumour number	3.046 (1.795–5.170)	**<0.001**	—	—
Tumour differentiation	2.081 (1.408–3.077)	**<0.001**	2.254 (1.462–3.476)	**<0.001**
Myosteatosis	1.269 (0.674–2.391)	0.460	—	—
SMRA reduction	0.967 (0.569–1.645)	0.902		
Muscle loss	2.261 (1.347–3.794)	**0.002**	2.286 (1.358–3.849)	**0.002**
Recurrence‐free survival				
Age at transplant	0.975 (0.951–1.000)	**0.046**	—	—
BMI	0.956 (0.889–1.029)	0.235	—	—
Cirrhosis	1.438 (0.526–3.933)	0.479	—	—
Hepatitis B	0.992 (0.363–2.712)	0.987	—	—
Child–Pugh class	1.014 (0.757–1.358)	0.926		
Ascites	1.088 (0.701–1.689)	0.707		
Variceal bleeding	0.521 (0.211–1.288)	0.158		
Hepatic encephalopathy	0.505 (0.070–3.629)	0.497		
MELD score	1.001 (0.983–1.018)	0.945	—	—
ABO compatibility	0.439 (0.242–0.797)	**0.007**	—	—
CIT	0.999 (0.924–1.080)	0.974	—	—
Operative time	1.141 (1.007–1.293)	**0.039**	—	—
Blood loss	1.622 (1.046–2.515)	**0.031**	—	—
Preoperative AFP level	1.780 (1.390–2.279)	**<0.001**	1.429 (1.107–1.843)	**0.006**
Tumour size	3.976 (2.531–6.245)	**<0.001**	3.653 (2.303–5.792)	**<0.001**
Tumour number	3.591 (2.278–5.661)	**<0.001**	—	—
Tumour differentiation	1.900 (1.376–2.623)	**<0.001**	1.842 (1.268–2.676)	**0.001**
Myosteatosis	1.177 (0.680–2.035)	0.561	—	—
SMRA reduction	1.041 (0.657–1.648)	0.865		
Muscle loss	2.053 (1.317–3.201)	**0.001**	2.219 (1.418–3.471)	**<0.001**

*Note*: Values in bold represent statistically significant differences.

Abbreviations: AFP, α‐fetoprotein; BMI, body mass index; CI, confidence interval; CIT, cold ischaemia time; HR, hazard ratio; MELD, Model for End‐Stage Liver Disease; SMRA, skeletal muscle radiodensity.

### Peri‐transplant muscle assessment: Mass, radiodensity and loss

**Figure 5:**
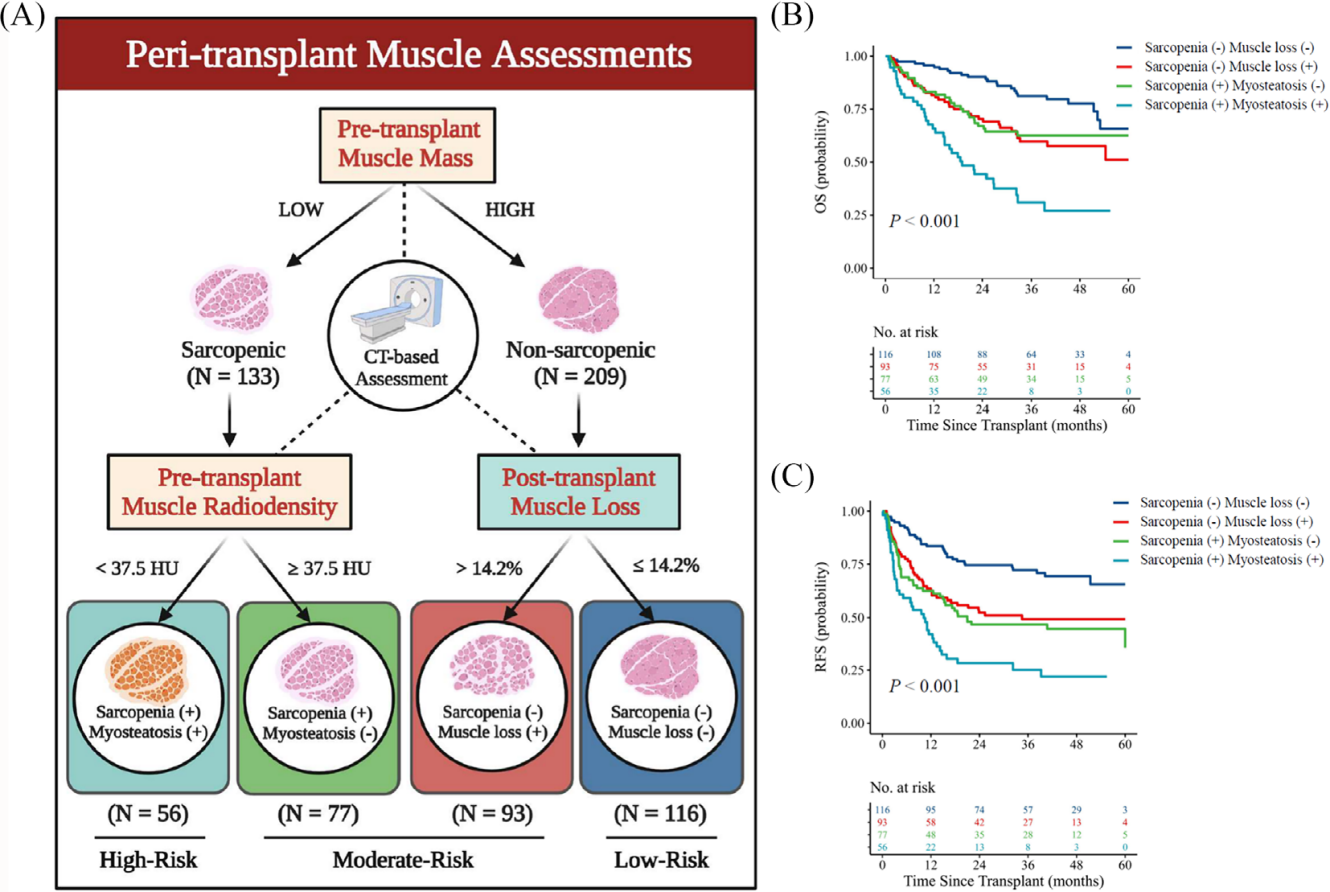
Peri‐transplant muscle assessment: mass, radiodensity, and loss. (*A*) Peri‐transplant muscle assessments included three aspects: muscle mass, muscle radiodensity and muscle loss. Four populations with diverse patient outcomes were identified based on the above assessment strategy. (*B,C*) OS and RFS curves for the four populations with different muscle status using log‐rank tests. CT, computed tomography; OS, overall survival; RFS, recurrence‐free survival; SMRA, skeletal muscle radiodensity.

In the preceding sections, we discussed the significance of sarcopenia, myosteatosis and post‐transplant muscle loss in patients undergoing DDLT for HCC. To summarize, 342 recipients with available CT images were further grouped according to an assumed workflow, as given in *Figure* *5* *A*. Non‐sarcopenic recipients with low muscle loss had the best prognosis among the above four groups and were considered low risk. Sarcopenic recipients with myosteatosis had the worst outcomes and were deemed as high risk. The other two groups were ranked as the moderate‐risk populations. The survival curves are depicted in *Figure* *5* *B,C*.

## Discussion

To our knowledge, this is the first multicentre clinical study to investigate the correlation between skeletal muscle status and the prognosis of LT for HCC, with the primary outcomes of RFS and OS. A previous study uncovered a crosstalk between skeletal muscle and hepatocellular carcinoma, suggesting that sarcopenia may promote tumour recurrence.^21^ However, this finding lacked validation in a large clinical cohort. The present study addressed this gap by including tumour recurrence as one of the observed outcomes. The skeletal muscle status in the study included sarcopenia, myosteatosis and post‐LT muscle loss. The study identified the skeletal muscle subgroup, based on the occurrence of sarcopenia and myosteatosis, as independent risk factors for adverse OS and RFS in male recipients with HCC after LT. Diverging from previous studies that focused on the cross‐sectional status of sarcopenia, the study evaluated changes in muscle mass during the peri‐transplant period and found its impact on the prognosis of non‐sarcopenic patients. This appears significant for clinical diagnosis and treatment, as non‐sarcopenic patients, traditionally perceived to have a favourable prognosis, have not been given much attention in the past. Finally, the study established a simplified workflow for both sarcopenic and non‐sarcopenic recipients, hoping to differentiate recipients with distinct prognostic risks in clinical diagnosis and treatment.

LT for HCC is a radical treatment option for patients with early‐stage disease and accounts for up to 40% of all LT performed worldwide.^22^, ^23^, ^24^ Despite careful patient selection, many recipients still suffer tumour recurrence, resulting in high mortality rates.^25^, ^26^ To improve the outcome of LT for HCC, a series of candidate selection criteria that mainly focus on tumour‐related factors have been proposed.^27^, ^28^, ^29^ Other factors that may contribute to post‐LT outcomes have been considered, such as donor characteristics, recipient inflammatory status and explant pathology.^22^, ^30^ The question is can we go further? We recently participated in a global study that investigated the prognostic value of muscle mass in patients who received LT for HCC beyond the Milan criteria, and the result revealed the added prognostic value of muscle mass.^15^ Besides muscle mass, we noticed that muscle radiodensity is another critical issue in reflecting muscle status. We also observed that many patients experienced significant muscle loss after LT, often accompanied by muscle weakness during physical examination and limb thinning at the bedside. Inspired by the observations mentioned above, we conducted this study to explain the patient's prognosis from the perspective of skeletal muscle and hoped to reconsider the boundaries of LT for HCC.

Muscle quality has always been an attractive topic in multiple diseases.^31^, ^32^ Evidence suggests that using myosteatosis to evaluate muscle quality may also have favourable prognostic value.^13^ In this study, myosteatosis is associated with poor outcomes after LT in male patients. The above‐mentioned adverse survival outcomes may be partly attributed to myosteatotic patients experiencing more postoperative complications, aligning with previous reports.^33^ From a mechanistic standpoint, the increased accumulation of intramuscular fat is associated with a pro‐inflammatory microenvironment, leading to heightened oxidative stress, increased graft injury, and impairment of immune function.^34^, ^35^ In this study consisting of HCC patients, the inherent systemic energy metabolism disturbances may be one of the factors contributing to the occurrence of myosteatosis. Additionally, research indicates that tumour tissues can secrete a range of pro‐cachectic cytokines, which may act on target organs such as skeletal muscles, influencing their energy metabolism.^36^ To investigate whether the prognostic implication of myosteatosis applies to sarcopenic patients, we conducted subgroup analyses and were surprised to find that myosteatosis could predict poor OS and RFS only in sarcopenic recipients. The observed differences suggest a potential connection between sarcopenia and myosteatosis, in which sarcopenia seems to act as a precondition part for adverse prognosis. Overall, our results suggested that neither muscle mass nor radiodensity should be ignored in the systemic evaluation.

LT is rather destructive for the patients' general conditions, resulting in a second hit for the HCC patients with pre‐existing cirrhosis. Some patients experienced severe muscle loss and became extremely weak after surgery. To explore the role of muscle loss in predicting post‐LT outcomes, we conducted dynamic analyses to demonstrate the significance of muscle loss in predicting outcomes. To avoid overdiagnosis, we did not use the preoperative criteria to diagnose sarcopenia.^37^ Instead, we used dynamic muscle loss by comparing pre‐ and post‐transplant SMI. Interestingly, the non‐sarcopenic recipients tended to suffer a remarkable decline in SMI after transplantation while in the sarcopenic group, the muscle loss might be concealed by low preoperative SMI. Therefore, we separately investigated the prognostic value of muscle loss in the sarcopenic and non‐sarcopenic groups. For non‐sarcopenic recipients, higher muscle loss indicated poorer outcomes. In other words, it is far from enough to rely solely on preoperative evaluation of muscle status without considering the peri‐transplant muscle loss. Thus, we introduced a simple workflow, hoping to perform individualized peri‐transplant management in clinical practice.

There are several limitations to this study. The study focuses on analysing Chinese male HCC patients, the majority of whom had hepatitis B and cirrhosis. Taking into account the differences in body size and nutritional levels between Eastern and Western populations, external validity in Western populations may be quite challenging. Due to the epidemiological characteristics of HCC, males accounted for about 89% of this cohort. We focus on the males in this study while the impact of body compositions on prognosis in female HCC recipients needs further confirmation in a larger cohort. In addition, there may be selection bias due to the lack of postoperative CT scans in partial patients. Due to the inconsistency in the postoperative CT follow‐up times among different medical centres, we are unable to analyse the muscle loss in all patients. In addition to routine follow‐ups, early postoperative complications can also result in the bias of CT collection, so the data should be interpreted with caution. We are ready to design a prospective study that may minimize the bias. Moreover, variations in CT parameters among different centres may limit the generalizability of the conclusions drawn in the article.

In conclusion, this study provides a peri‐transplant skeletal muscle assessment workflow for male HCC patients undergoing LT. For confirmed sarcopenic patients, myosteatosis needs to be monitored and addressed, as its occurrence is accompanied by a further decline in survival rates. In non‐sarcopenic patients, excessive muscle loss is correlated with adverse outcomes, emphasizing the importance of post‐transplant monitoring and nutritional support.

## Conflict of interest statement

Di Lu, Zhihang Hu, Hao Chen, Abid Ali Khan, Qingguo Xu, Zuyuan Lin, Huigang Li, Jianyong Zhuo, Chiyu He, Li Zhuang, Zhe Yang, Siyi Dong, Jinzhen Cai, Shusen Zheng and Xiao Xu declare that they have no known competing financial interests or personal relationships that could have appeared to influence the work reported in this paper.

## Supporting information


**Figure S1** Dynamic evaluation of muscle mass and radiodensity among the postoperative period. (A) No significant correlation was observed between POD and the muscle loss using Spearman's rank correlation analysis (*P* = 0.436). (B) No significant correlation was observed between POD and the SMRA reduction using Spearman's rank correlation analysis (*P* = 0.830). SMRA, skeletal muscle radiodensity; POD, postoperative day.
**Figure S2** The variation of SMRA after LT. SMRA decreased significantly after LT using Paired Samples t‐test (*P* < 0.001). SMRA, skeletal muscle radiodensity; LT, liver transplantation.

**Table S1.** Patient characteristics in the dynamic population (*N* = 342)
**Table S2.** Comparisons of body parameters between the Eastern and Western populations with cancer
**Table S3.** Body Compositions According to International and Chinese BMI Categories
**Table S4.** Body composition associated with poor recurrence‐free survival and overall survival in male and female recipients
**Table S5.** Characteristics in the different subgroups
**Table S6.** Precision error and least significant change of the skeletal muscle area and radiodensity
**Table S7.** Variables Associated with Poor Outcomes by Multivariate Cox Regression in Sarcopenic Patients (*N* = 133)
**Table S8.** Characteristics in Non‐sarcopenic Patients with Different Levels of Muscle Loss

## Data Availability

The data that support the findings of this study are available from the corresponding author, Xiao Xu, upon reasonable request.
