# Supplementary material for: Myosteatosis and muscle loss impact liver transplant outcomes in male patients with hepatocellular carcinoma
Source: J Cachexia Sarcopenia Muscle. 2024 Aug 27;15(5):2071–83. doi: 10.1002/jcsm.13554 (PMC11446693; doi:10.1002/jcsm.13554)
Supplement: Supplementary file 1 — Figure S1 Dynamic evaluation of muscle mass and radiodensity among the postoperative period. (A) No significant correlation was observed between POD and the muscle loss using Spearman's rank correlation analysis (P = 0.436). (B) No significant correlation was observed between POD and the SMRA reduction using Spearman's rank correlation analysis (P = 0.830). SMRA, skeletal muscle radiodensity; POD, postoperative day. Figure S2 The variation of SMRA after LT. SMRA decreased significantly after LT using Paired Samples t‐test (P < 0.001). SMRA, skeletal muscle radiodensity; LT, liver transplantation. [file JCSM-15-2071-s001.docx]

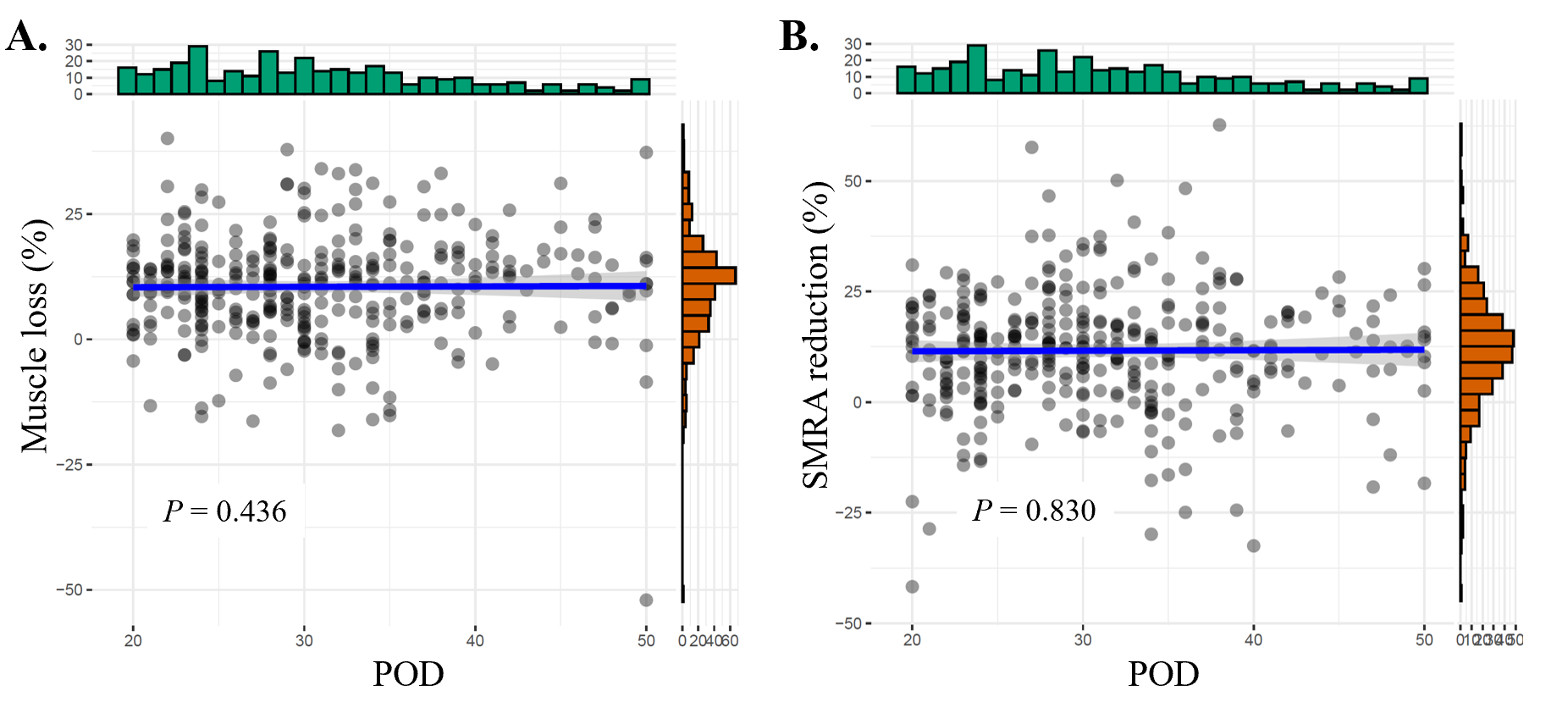


**Figure S1 Dynamic evaluation of muscle mass and radiodensity among the postoperative period.** (A) No significant correlation was observed between POD and the muscle loss using Spearman’s rank correlation analysis (*P* = 0.436). (B) No significant correlation was observed between POD and the SMRA reduction using Spearman’s rank correlation analysis (*P* = 0.830). SMRA, skeletal muscle radiodensity; POD, postoperative day.


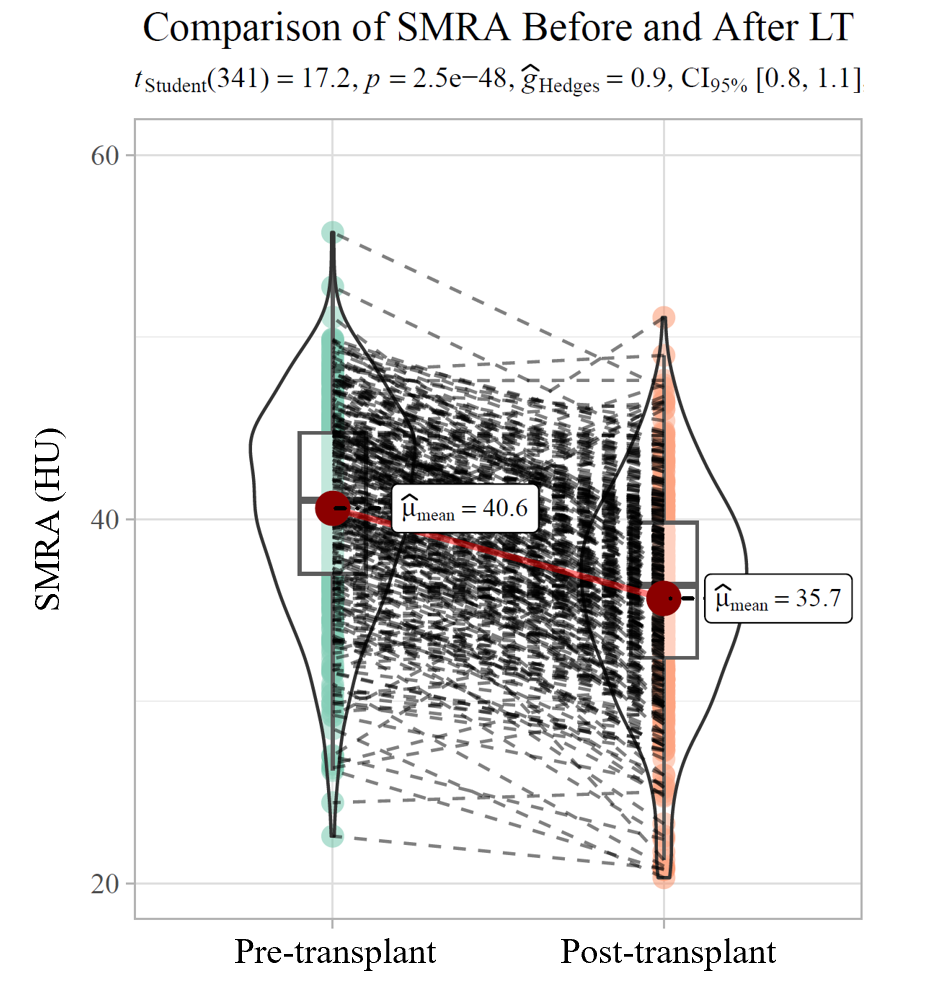


**Figure S2 The variation of SMRA after LT.** SMRA decreased significantly after LT using Paired Samples t-test (*P* < 0.001). SMRA, skeletal muscle radiodensity; LT, liver transplantation.
