# Supplementary material for: Myosteatosis and muscle loss impact liver transplant outcomes in male patients with hepatocellular carcinoma
Source: J Cachexia Sarcopenia Muscle. 2024 Aug 27;15(5):2071–83. doi: 10.1002/jcsm.13554 (PMC11446693; doi:10.1002/jcsm.13554)
Supplement: Supplementary file 2 — Table S1. Patient characteristics in the dynamic population (N = 342) Table S2. Comparisons of body parameters between the Eastern and Western populations with cancer Table S3. Body Compositions According to International and Chinese BMI Categories Table S4. Body composition associated with poor recurrence‐free survival and overall survival in male and female recipients Table S5. Characteristics in the different subgroups Table S6. Precision error and least significant change of the skeletal muscle area and radiodensity Table S7. Variables Associated with Poor Outcomes by Multivariate Cox Regression in Sarcopenic Patients (N = 133) Table S8. Characteristics in Non‐sarcopenic Patients with Different Levels of Muscle Loss [file JCSM-15-2071-s002.docx]

**Table S1. Patient characteristics in the dynamic population (N=342)**

| **Characteristics** |  |
| --- | --- |
| Age at transplant, median (IQR), yr | 53.0 (47.0-59.0) |
| BMI, median (IQR), kg/m^2^ | 23.5 (21.8-25.8) |
| Hepatitis B, no. (%) | 319 (93.3) |
| Cirrhosis, no. (%) | 314 (91.8) |
| MELD score, median (IQR) | 31.0 (16.0-39.0) |
| Child-Pugh class, no. (%) |  |
| A (5-6) | 49 (14.3) |
| B (7-9) | 78 (22.8) |
| C (10-15) | 215 (62.9) |
| Identical ABO compatibility, no. (%) | 305 (89.2) |
| CIT, median (IQR), h | 8.0 (7.0-10.0) |
| Operative time, median (IQR), h | 5.3 (4.5-6.3) |
| Intraoperative blood loss > 1 L, no. (%) | 165 (48.2) |
| Tumor number > 3, no. (%) | 87 (25.4) |
| Sum of tumor diameters > 8 cm, no. (%) | 141 (41.2) |
| Last pretransplant α-fetoprotein, no. (%) |  |
| ≤ 100 ng/mL | 203 (59.4) |
| 100-400 ng/mL | 43 (12.6) |
| > 400 ng/mL | 96 (28.1) |
| Tumor differentiation, no. (%) |  |
| Complete necrosis/no viable tumor | 25 (7.3) |
| Well differentiated | 23 (6.7) |
| Moderately differentiated | 190 (55.6) |
| Poorly differentiated | 104 (30.4) |
| SMI, median (IQR), cm^2^/m^2^ | 48.7 (43.5-53.6) |
| SMRA, median (IQR), HU | 41.0 (37.0-44.8) |
| Sarcopenia, no. (%) | 133 (38.9) |
| Myosteatosis, no. (%) | 95 (27.8) |
| Early complications, no. (%) | 66 (19.3) |
| 90day-mortality, no. (%) | 18 (5.3) |

**Table S2. Comparisons of body parameters between the Eastern and Western populations with cancer**

| **Variable** | **The Western Population*** | |  | **The Eastern Population (Ours)** | |
| --- | --- | --- | --- | --- | --- |
|  | **Men (N = 828)** | **Women (N = 645)** |  | **Men (N = 673)** | **Women (N = 83)** |
| Height, Mean (SD), cm | 175.5 (6.9) | 161.1 (6.9) |  | 171.0 (5.2) | 160.0 (5.0) |
| Weight, Mean (SD), kg | 80.1 (16.2) | 65.3 (15.7) |  | 70.0 (11.0) | 58.7 (9.9) |
| BMI, Mean (SD), kg/m^2^ | 26.0 (4.9) | 25.1 (5.8) |  | 23.9 (3.3) | 22.9 (3.6) |
| BMI category, No. (%), kg/m^2^ |  |  |  |  |  |
| > 30.0 | 67 (8.1) | 114 (17.7) |  | 34 (5.1) | 3 (3.6) |
| 25.0 to 29.9 | 300 (36.2) | 236 (36.6) |  | 182 (27.0) | 14 (16.9) |
| 20.0 to 24.9 | 328 (39.6) | 183 (28.4) |  | 393 (58.4) | 49 (59.0) |
| < 20.0 | 133 (16.1) | 112 (17.3) |  | 71 (10.5) | 17 (20.5) |

* Reference: Martin L, et al. J Clin Oncol. 2013.

| **Table S3. Body Compositions According to International and Chinese BMI Categories** | | | |
| --- | --- | --- | --- |
| **S3A: International BMI Category** | | | |
| **Variable** | **All Patients** | | ***P* value** |
|  | **BMI < 25 kg/m^2^ (N = 523)** | **BMI** ≥ **25 kg/m^2^ (N = 233)** |  |
| SMI, Mean (SD), cm^2^/m^2^ | 45.3 (7.6) | 53.9 (7.4) | **< 0.001** |
| SMRA, Mean (SD), HU | 40.5 (5.9) | 39.6 (6.2) | 0.054 |
|  | | | |
| **S3B: Chinese BMI Category*** | | | |
| **Variable** | **All Patients** | | ***P* value** |
|  | **BMI < 24 kg/m^2^ (N = 436)** | **BMI** ≥ **24 kg/m^2^ (N = 320)** |  |
| SMI, Mean (SD), cm^2^/m^2^ | 44.6 (7.5) | 52.5 (7.6) | **< 0.001** |
| SMRA, Mean (SD), HU | 40.5 (5.8) | 39.8 (6.2) | 0.124 |

* Reference: Gao M, et al. PLoS Med. 2020.

**Table S4. Body composition associated with poor recurrence-free survival and overall survival in male and female recipients**

| **S4A: Recurrence-free Survival** | |  |  |  |  |
| --- | --- | --- | --- | --- | --- |
| **Variable** | **Male** | |  | **Female** | |
|  | **HR (95% CI)** | ***P* value** |  | **HR (95% CI)** | ***P* value** |
| SMI, cm^2^/m^2^ | 0.964 (0.949-0.979) | **<0.001** |  | 0.964 (0.910-1.022) | 0.219 |
| SMRA, HU | 0.968 (0.950-0.988) | **0.001** |  | 1.016 (0.958-1.079) | 0.594 |
|  |  |  |  |  |  |
| **S4B: Overall Survival** |  |  |  |  |  |
| **Variable** | **Male** | |  | **Female** | |
|  | **HR (95% CI)** | ***P* value** |  | **HR (95% CI)** | ***P* value** |
| SMI, cm^2^/m^2^ | 0.956 (0.939-0.974) | **<0.001** |  | 0.992 (0.933-1.055) | 0.799 |
| SMRA, HU | 0.953 (0.932-0.976) | **<0.001** |  | 1.041 (0.969-1.119) | 0.274 |

**Table S5. Characteristics in the different subgroups**

| **Characteristics** | **Non-sarcopenic (N = 406 )** | **Sarcopenic** | | ***P* value** |
| --- | --- | --- | --- | --- |
|  |  | **Non-myosteatotic (N = 159 )** | **Myosteatotic (N = 108 )** |  |
| Age at transplant, median (IQR), yr | 53.0 (47.0-58.0) | 53.0 (46.5-59.0) | 56.0 (50.0-63.0) | **<0.001** |
| BMI, median (IQR), kg/m^2^ | 24.0 (22.5-26.5) | 21.8 (20.5-24.2) | 23.4 (21.6-23.8) | **<0.001** |
| Hepatitis B | 375 (92.4) | 159 (100.0) | 90 (83.3) | **<0.001** |
| Cirrhosis | 378 (93.1) | 133 (83.6) | 104 (96.3) | **<0.001** |
| MELD score, median (IQR) | 23.0 (10.0-37.0) | 22.0 (10.0-37.0) | 27.5 (14.8-35.3) | 0.402 |
| Child-Pugh class, no. (%) |  |  |  | **0.003** |
| A (5-6) | 99 (24.4) | 44 (27.7) | 12 (11.1) |  |
| B (7-9) | 130 (32.0) | 37 (23.3) | 32 (29.6) |  |
| C (10-15) | 177 (43.6) | 78 (49.1) | 64 (59.3) |  |
| Ascites, no. (%) | 193 (47.5) | 81 (50.9) | 71 (65.7) | **0.003** |
| Variceal bleeding, no. (%) | 46 (11.3) | 24 (15.1) | 11 (10.2) | 0.378 |
| Hepatic encephalopathy, no. (%) | 9 (2.2) | 8 (5.0) | 3 (2.8) | 0.242 |
| Identical ABO compatibility, no. (%) | 373 (91.9) | 146 (91.8) | 97 (89.8) | 0.783 |
| CIT, median (IQR), h | 8.0 (5.9-9.5) | 7.6 (5.1-9.1) | 8.0 (6.0-9.1) | 0.061 |
| Operative time, median (IQR), h | 5.7 (4.8-7.0) | 5.6 (4.7-6.7) | 6.0 (5.1-7.3) | 0.130 |
| Intraoperative blood loss > 1 L, no. (%) | 137 (33.7) | 68 (42.8) | 60 (55.6) | **<0.001** |
| Tumor number > 3, no. (%) | 79 (19.5) | 43 (27.0) | 29 (26.9) | 0.074 |
| Sum of tumor diameters > 8 cm, no. (%) | 136 (33.5) | 69 (43.4) | 51 (47.2) | **0.009** |
| Last pretransplant α-fetoprotein, no. (%) |  |  |  | **0.018** |
| ≤ 100 ng/mL | 270 (66.5) | 85 (53.5) | 61 (56.5) |  |
| 100-400 ng/mL | 50 (12.3) | 26 (16.4) | 12 (11.1) |  |
| > 400 ng/mL | 86 (21.2) | 48 (30.2) | 35 (32.4) |  |
| Tumor differentiation, no. (%) |  |  |  | 0.619 |
| Complete necrosis/no viable tumor | 41 (10.1) | 13 (8.2) | 6 (5.6) |  |
| Well differentiated | 23 (5.7) | 9 (5.7) | 5 (4.6) |  |
| Moderately differentiated | 214 (52.7) | 92 (57.9) | 57 (52.8) |  |
| Poorly differentiated | 128 (31.5) | 45 (28.3) | 40 (37.0) |  |
| SMI, median (IQR), cm^2^/m^2^ | 52.5 (48.7-57.3) | 42.5 (39.2-44.8) | 41.9 (36.7-45.5) | **<0.001** |
| SMRA, median (IQR), HU | 42.7 (38.7-45.4) | 42.2 (40.1-45.1) | 34.4 (30.9-36.0) | **<0.001** |

**Table S6. Precision error and least significant change of the skeletal msucle area and radiodensity**

| **S6A: Observer A** |  |  |
| --- | --- | --- |
| **Parameters** | **%CV** | **LSC_%CV_ (95% CI)** |
| Muscle area, cm^2^ | 0.540 | 1.500 |
| Muscle radiodensity, HU | 0.249 | 0.690 |
|  |  |  |
| **S6B: Observer B** |  |  |
| **Parameters** | **%CV** | **LSC_%CV_ (95% CI)** |
| Muscle area, cm^2^ | 0.645 | 1.787 |
| Muscle radiodensity, HU | 0.355 | 0.983 |

%CV, % coefficient of variability; LSC, least significant change; LSC %CV, least significant change for the for the root-mean -square percent coefficient of variation.

**Table S7. Variables Associated with Poor Outcomes by Multivariate Cox Regression in Sarcopenic Patients (N = 133)**

| **S7A: Overall Survival** |  |  |  |  |  |
| --- | --- | --- | --- | --- | --- |
| **Variable** | **Univariable** | |  | **Multivariable** | |
|  | **HR (95% CI)** | ***P* value** |  | **HR (95% CI)** | ***P* value** |
| Age at transplant | 0.992 (0.967-1.018) | 0.537 |  | - | - |
| BMI | 0.981 (0.911-1.056) | 0.613 |  | - | - |
| Cirrhosis | 0.916 (0.436-1.922) | 0.816 |  | - | - |
| Hepatitis B | 1.046 (0.419-2.607) | 0.924 |  | - | - |
| Child-Pugh class | 1.135 (0.788-1.635) | 0.495 |  |  |  |
| Ascites | 1.645 (0.961-2.817) | **0.070** |  |  |  |
| Variceal bleeding | 0.914 (0.394-2.121) | 0.835 |  |  |  |
| Hepatic encephalopathy | 1.021 (0.320-3.257) | 0.971 |  |  |  |
| MELD score | 1.010 (0.988-1.033) | 0.369 |  | - | - |
| ABO compatibility | 0.415 (0.216-0.795) | **0.008** |  | - | - |
| CIT | 1.017 (0.925-1.118) | 0.733 |  | - | - |
| Operative time | 1.012 (0.828-1.237) | 0.907 |  | - | - |
| Blood loss | 1.607 (0.958-2.696) | **0.072** |  | - | - |
| Preoperative AFP level | 1.730 (1.323-2.262) | **<0.001** |  | 1.895 (1.442-2.491) | **<0.001** |
| Tumor size | 1.905 (1.157-3.136) | **0.011** |  | 2.154 (1.280-3.625) | **0.004** |
| Tumor number | 1.483 (0.892-2.465) | 0.128 |  | - | - |
| Tumor differentiation | 1.858 (1.261-2.737) | **0.002** |  | - | - |
| Myosteatosis | 2.396 (1.457-3.941) | **<0.001** |  | 3.496 (2.055-5.946) | **<0.001** |
| SMRA decrease | 1.394 (0.836-2.324) | 0.203 |  |  |  |
| Muscle loss | 1.206 (0.614-2.370) | 0.587 |  | - | - |
|  |  |  |  |  |  |
| **S7B: Recurrence-free Survival** | |  |  |  |  |
| **Variable** | **Univariable** | |  | **Multivariable** | |
|  | **HR (95% CI)** | ***P* value** |  | **HR (95% CI)** | ***P* value** |
| Age at transplant | 0.986 (0.964-1.008) | 0.206 |  | - | - |
| BMI | 0.981 (0.916-1.049) | 0.571 |  | - | - |
| Cirrhosis | 0.649 (0.351-1.200) | 0.168 |  | - | - |
| Hepatitis B | 1.271 (0.554-2.919) | 0.571 |  | - | - |
| Child-Pugh class | 1.007 (0.741-1.369) | 0.962 |  |  |  |
| Ascites | 1.410 (0.892-2.231) | 0.142 |  |  |  |
| Variceal bleeding | 0.859 (0.414-1.783) | 0.684 |  |  |  |
| Hepatic encephalopathy | 1.379 (0.557-3.411) | 0.487 |  |  |  |
| MELD score | 1.009 (0.989-1.029) | 0.376 |  | - | - |
| ABO compatibility | 0.499 (0.270-0.923) | **0.027** |  | - | - |
| CIT | 1.007 (0.929-1.093) | 0.858 |  | - | - |
| Operative time | 0.997 (0.844-1.178) | 0.971 |  | - | - |
| Blood loss | 1.488 (0.951-2.327) | **0.082** |  | - | - |
| Preoperative AFP level | 1.624 (1.288-2.048) | **<0.001** |  | 1.686 (1.328-2.142) | **<0.001** |
| Tumor size | 2.041 (1.322-3.152) | **0.001** |  | 2.026 (1.297-3.164) | **0.002** |
| Tumor number | 1.700 (1.095-2.641) | **0.018** |  | - | - |
| Tumor differentiation | 1.532 (1.118-2.101) | **0.008** |  | - | - |
| Myosteatosis | 1.793 (1.162-2.767) | **0.008** |  | 2.459 (1.556-3.888) | **<0.001** |
| SMRA decrease | 1.213 (0.769-1.913) | 0.406 |  |  |  |
| Muscle loss | 0.963 (0.522-1.776) | 0.904 |  | - | - |

**Table S8. Characteristics in Non-sarcopenic Patients with Different Levels of Muscle Loss**

| **Characteristics** | **Non-sarcopenia (N=209)** | | ***P* value** |
| --- | --- | --- | --- |
|  | **Low Muscle Loss (N = 116 )** | **High Muscle Loss (N = 93 )** |  |
| Age at transplant, median (IQR), yr | 54.0 (48.0-61.0) | 52.0 (46.0-57.0) | 0.057 |
| BMI, median (IQR), kg/m2 | 23.7 (22.5-25.7) | 24.2 (22.7-27.3) | 0.126 |
| Hepatitis B | 109 (94.0) | 88 (94.6) | 0.839 |
| Cirrhosis | 109 (94.0) | 87 (93.5) | 0.901 |
| Ascites, no. (%) | 58 (50.0) | 53 (57.0) | 0.314 |
| Variceal bleeding, no. (%) | 13 (11.2) | 8 (8.6) | 0.534 |
| Hepatic encephalopathy, no. (%) | 2 (1.7) | 2 (2.2) | 1.000 |
| MELD score, median (IQR) | 29.5 (11.0-39.0) | 30.0 (14.0-38.0) | 0.817 |
| Child-Pugh class, no. (%) |  |  | 0.888 |
| A (5-6) | 20 (17.2) | 14 (15.1) |  |
| B (7-9) | 30 (25.9) | 26 (28.0) |  |
| C (10-15) | 66 (56.9) | 53 (57.0) |  |
| Identical ABO compatibility, no. (%) | 108 (93.1) | 79 (84.9) | 0.056 |
| CIT, median (IQR), h | 8.0 (7.0-10.0) | 8.0 (7.0-10.0) | 0.817 |
| Operative time, median (IQR), h | 5.1 (4.4-6.2) | 5.2 (4.7-6.7) | 0.400 |
| Intraoperative blood loss > 1 L, no. (%) | 43 (37.1) | 44 (47.3) | 0.135 |
| Tumor number > 3, no. (%) | 21 (18.1) | 22 (23.7) | 0.324 |
| Sum of tumor diameters > 8 cm, no. (%) | 38 (32.8) | 37 (39.8) | 0.293 |
| Last pretransplant α-fetoprotein, no. (%) |  |  | 0.316 |
| ≤ 100 ng/mL | 75 (64.7) | 62 (66.7) |  |
| 100-400 ng/mL | 19 (16.4) | 9 (9.7) |  |
| > 400 ng/mL | 22 (19.0) | 22 (23.7) |  |
| Tumor differentiation, no. (%) |  |  | 0.841 |
| Complete necrosis/no viable tumor | 11 (9.5) | 7 (7.5) |  |
| Well differentiated | 8 (6.9) | 8 (8.6) |  |
| Moderately differentiated | 63 (54.3) | 47 (50.5) |  |
| Poorly differentiated | 34 (29.3) | 31 (33.3) |  |
| SMI, median (IQR), cm2/m2 | 51.5 (48.4-55.7) | 53.7 (49.4-58.3) | **0.016** |
| SMRA, median (IQR), HU | 42.6 (39.5-44.9) | 42.2 (37.2-45.8) | 0.418 |
| ICU stay, median (IQR), hours | 279 (195-330) | 303 (192-389) | 0.067 |
